# CircMAN1A2 promotes vasculogenic mimicry of nasopharyngeal carcinoma cells through upregulating ERBB2 via sponging miR-940

**DOI:** 10.32604/or.2022.027534

**Published:** 2023-01-31

**Authors:** HUAQING MO, JINGYI SHEN, YUXIAO ZHONG, ZENAN CHEN, TONG WU, YANYU LV, YANYAN XIE, YANRONG HAO

**Affiliations:** Cancer Center, The People’s Hospital of Guangxi Zhuang Autonomous Region, Guangxi Academy of Medical Sciences, Nanning, 530021, China

**Keywords:** MiR-940, circMAN1A2, ERBB2, Vasculogenic mimicry, Nasopharyngeal carcinoma, PI3K/AKT/mTOR signaling pathway

## Abstract

Nasopharyngeal carcinoma (NPC) is the most prevalent human primary malignancy of the head and neck, and the presence of vasculogenic mimicry (VM) renders anti-angiogenic therapy ineffective and poorly prognostic. However, the underlying mechanisms are unclear. In the present study, we used miR-940 silencing and overexpression for *in vitro* NPC cell EdU staining, wound healing assay and 3D cell culture assay, and *in vivo* xenograft mouse model and VM formation to assess miR-940 function. We found that ectopic miR-940 expression reduced NPC cell proliferation, migration and VM, as well as tumorigenesis *in vivo*. By bioinformatic analysis, circMAN1A2 was identified as a circRNA that binds to miR-940. Mechanistically, we confirmed that circMAN1A2 acts as a sponge for miR-940, impairs the inhibitory effect of miR-940 on target ERBB2, and then activates the PI3K/AKT/mTOR signaling pathway using RNA-FISH, dual luciferase reporter gene and rescue analysis assays. In addition, upregulation of ERBB2 expression is associated with clinical staging and poor prognosis of NPC. Taken together, the present findings suggest that circMAN1A2 promotes VM formation and progression of NPC through miR-940/ERBB2 axis and further activates the PI3K/AKT/mTOR pathway. Therefore, circMAN1A2 may become a biomarker and therapeutic target for anti-angiogenic therapy in patients with nasopharyngeal carcinoma.

## Introduction

Nasopharyngeal carcinoma (NPC) originates from the epithelial cells of the nasopharynx and is highly aggressive and metastatic [1]. The formation of NPC involves various biological processes such as proliferation, migration and especially angiogenesis [2]. More importantly, tumor angiogenesis is an important cause of NPC metastasis [3]. However, the clinical efficacy of anti-angiogenic drugs in NPC has been far below expectations to date [4–6], presumably as the underlying mechanisms of angiogenesis have not been elucidated. Notably, Maniotis et al. [7] discovered in 1999 that the tubular structures formed by cancer cells also supply blood to the tumor and easily lead to distant transfer via blood flow [8], which may explain the failure of the drug to prevent metastasis. Although VM has been observed in NPC [9], the mechanism by which it occurs is unclear.

MicroRNAs (miRNAs) block gene transcription by binding directly to the 3′ untranslated region (3′-UTR) of the target mRNA, resulting in altered cellular function [10]. MiR-940 is dysregulated in a variety of cancers [11]. In certain cancers, miR-940 acts ontogenically by blocking malignant biological behavior [12–16]. Also, its expression has been associated with drug resistance and prognosis [17]. The finding that miR-940 was lower expressed in NPC tissues than in paraneoplastic tissues [18] inspired us to explore its function and regulatory mechanisms further.

Circular RNAs (circRNAs) are a class of RNA molecules characterized by covalently closed loops, commonly involved in tumorigenesis and development through spongy miRNAs [19,20]. Due to its stable and widespread expression, it is expected to be an ideal candidate biomarker for cancer diagnosis, treatment and prognosis [21,22]. Studies have shown that circMAN1A2 (hsa_circ_0000119) is dramatically upregulated in patients with solid tumors and has good clinical diagnostic value [23–26]. However, the function and mechanism of circMAN1A2 in tumors are unclear.

The ERBB2 gene (also known as HER2, neu), an important member of the EGFR family, has intrinsic tyrosine kinase activity [27]. Ectopic expression of ERBB2 has been identified in various tumors [28]. High expression of ERBB2 not only regulates malignant biological behavior but also enhances tumor radio-resistance [29], leading to metastasis and poorer treatment response [30,31]. Several studies suggest ERBB2 is an important molecule for VM formation [32,33], and further exploration of its influence and regulatory mechanism in VM of NPC brings new insights for targeted therapy.

In the current study, we identify a circMAN1A2 associated with VM formation, which promotes the development and progression of NPC as an oncogene, and explores its clinical potential as a biomarker and therapeutic target for VM.

## Materials and Methods

### Cell lines

5-8F was gifted by Professor Musheng Zeng (Sun Yat-sen University). NP69 and HK-1 were gifted by Professor Sai-Wah Tsao (University of Hong Kong). Human NPC cell lines (5-8F, HK-1) were cultured in RPMI 1640 medium (Gibco, NY, USA) containing 10% fetal bovine serum (Gibco). The immortalized nasopharyngeal epithelial cells NP69 were cultured in a keratinocyte-serum-free medium containing 5% bovine pituitary extract and recombinant epidermal growth factor (Gibco). All cells were incubated at 37°C and 5% CO_2_.

### Clinical data

NPC clinical data and ERBB2 expression were collected from the GEPIA database (http://gepia.cancer-pku.cn/).

### Cell transfection

Lentivirus-mediated miR-940 overexpression vector (LV-miR-940), miR-940 knockdown vector (sh-miR-940), ERBB2 overexpression vector (LV-ERBB2) and ERBB2 silencing vector (sh-ERBB2) were purchased from Gene (Shanghai, China) and cells were infected with MOI = 50. Plasmids pcDNA3.1(+)-circMAN1A2, si-circMAN1A2 and miR-940 mimics or inhibitors were purchased from GenePharma (Shanghai, China). NPC cell lines were transfected with 100 nmol of pcDNA3.1(+)-circMAN1A2, si-circMAN1A2, or miR-940 mimics into NPC cells at 70% confluence using Lipofectamine 3000 (Invitrogen, MA, USA). The inhibitor was transfected into NPC cells. The loci for these vectors are in Table 1.

**Table 1:** Target sequences of siRNAs

Plasmid sequences		Sequence
si-circMAN1A2	Sense Antisense	GAAAAGGGAAGAGGAAGAATT UUCUUCCUCUUCCCUUUUCTT
si-NC	Sense Antisense	UUCUCCGAACGUGUCACGUTT ACGUGACACGUUCGGAGAATT
miR-940 mimics	Sense Antisense	AAGGCAGGGCCCCCGCUCCCC GGAGCGGGGGCCCUGCCUUUU
miR-NC	Sense Antisense	UUCUCCGAACGUGUCACGUTT ACGUGACACGUUCGGAGAATT
miR-940 inhibitor	Sense	GGGGAGCGGGGGCCCUGCCUU
inhibitor NC	Sense	CAGUACUUUUGUGUAGUACAA

Note: siRNA or si-, small interfering RNA.

### Quantitative real-time PCR (RT-qPCR)

Total RNA was extracted from the cells using TRIzol reagent (Invitrogen, MA, USA). MiRNA was extracted from cell lines using the miRcute adsorption column method (Tiangen Biotech Co., Ltd, Beijing, China). CircRNA was extracted using the RNeasy Mini Kit kit (Qiagen, Hilden, Germany). Expression of miR-940 and U6 was performed using the All-in-OneTM miRNA qRT-PCR kit (GeneCopoeia, Inc., USA) in a 7500 system (Applied Biosystems, Thermo Fisher Scientific, USA) for reverse transcription and RT-qPCR reactions. CircMAN1A2 and GAPDH mRNA were detected using the TB Green Premix EX Taq^TM^ kit (Takara Bio Inc., Tokyo, Japan). ERBB2 mRNA was detected using FastKing RT Kit (With gDNase) reverse transcribed from cDNA and then evaluated using the Super Real Pre Mix Plus (SYBR Green) kit (Tiangen). MiR-940 primer (HmiRQ0845) was ordered from FulenGen (Guangzhou, China), and the U6 primer (CD201-0145) was ordered from Tiangen. Normalization was performed using U6 and GAPDH and quantified by the 2^−ΔΔCq^ method [34]. The primers are listed in Table 2.

**Table 2:** The sequences of primers for RT-qPCR

Gene		Primer sequences
CircMAN1A2	Sense Antisense	AGATGGGCAAAGATGGATTGA GCCTTCTCATGATCAGCTCG
ERBB2	Sense Antisense	TGTGACTGCCTGTCCCTACAA CCAGACCATAGCACACTCGG
GAPDH	Sense Antisense	GTGGAGTCCACTGGCGTCTT GTGCAGGAGGCATTGCTGAT

### RNA fluorescence in situ hybridization (RNA-FISH)

The expression and cellular localization of circMAN1A2 in 5-8F cells were detected by FISH analysis. CircMAN1A2 (5′-CY3-CGUUCUUCCUCUUCCCUUUUCAAAUUCACCAUUGC-3′) and U6 (5′-CY3-CACGAAUUUGCGUGUCAUCCUU-3′) FISH probes were designed and synthesized by GenePharma. Firstly, the 5-8F cells were seeded on the coverslip, and then fixed with 4% paraformaldehyde after crawling. The hybridization solution containing the probe was added dropwise and incubated at 37°C for 16 h. Finally, the nuclei were restained with DAPI. Images were acquired and analyzed for nuclear plasma fluorescence intensity using Image J software (National Institutes of Health, Sacaton, Arizona).

### Western blotting

Proteins in NPC cells and tissues were extracted using RIPA lysis buffer. Then, the proteins were denatured and separated on 8% SDS-PAGE gels and transferred to PVDF membranes. Proteins bands were incubated with primary antibodies (anti-ERBB2 (#4290S, CST), anti-PI3K (#4255, CST), anti-AKT (#9272, CST), anti-mTOR (#2983, CST) and anti-GAPDH (#5174, CST)) at 1:1000 dilution for 18 h at 4°C. Next, the bands were incubated for 2 h with the secondary antibody labeled with horseradish peroxidase (anti-HRP, CST, 1:3,000, #7074). Electrochemiluminescence reagents (Beyotime, Shanghai, China) were added to protein strips and imaged using an Odyssey Fc system (LI-COR Biosciences, Lincoln, NE, USA). Quantification was performed by ImageJ software.

### Dual-luciferase reporter gene assay

Based on the bioinformatics circNET database, we predicted the binding site between circMAN1A2 and miR-940. Based on bioinformatics software (TargetScan7.2, DIANA tools and miRWalk), we predicted the possible target genes of miR-940. CircMAN1A2 wild-type (circMAN1A2 3′UTR-WT) and mutant (circMAN1A2 3′UTR-Mut) sequences were constructed and inserted into the GV272 vector. ERBB2 wild-type (ERBB2 3′UTR-WT) and mutant (ERBB2 3′UTR-Mut) sequences were also constructed and inserted into the pmirGLO vector. The 100 nmol reporter plasmid was transfected into HEK-293T cells together with miR-940 mimics or miR-NC. Finally, firefly and sea kidney luciferase activities were assayed using the Dual-Luciferase Reporter System Kit (Promega Madison, Wisconsin, USA).

### EdU assay

Cell proliferation was assessed using the keyFluor488 Click-iT EdU Imaging Assay Kit (keyGEN Bio TECH, Nanjing, China). A total of 50 mM EdU was added to NPC cells placed at 37°C for 2 h. Then, cells were fixed and incubated for 30 min with the addition of Click-iT reaction buffer. Finally, Hoechst was added for all cell nucleic acid staining. Images were taken using the EVOS FL Auto Cell Imaging System (Life Technologies Corp Bothell, WA, USA). Cell proliferation rates were calculated using ImageJ software.

### Wound healing assay

NPC cells were prepared into a cell suspension of 3 × 10^5^ cells/ml. 70 μl/well of cell suspension was inoculated into the Culture-Insert (μ-Dish^35mm,high^, Ibidi, Martinsried, Germany), and cells were removed with forceps after growing over the insert area. The insert was used to produce a scratch 500 μm in width. The culture was continued by adding a serum-free medium. Images were acquired with an inverted microscope (Olympus, Japan) at 0 and 24 h.

### In vitro VM tube formation assay

VM of NPC cells was assessed by 3D cell culture assays. A total of 10 μl of Matrigel (BD Biosciences, Bedford, MA, USA) was added to each well of an angiogenic slide (μ-slide, Ibidi, Martinsried, Germany) using a pre-cooled gun tip. The slides were incubated at 37°C, 5% CO_2_ for 30 min. Next, 5 × 10^4^ cells were added and then placed in the incubator for 12 h to 18 h. VM of cells was observed using an IX71 fluorescence microscope (Olympus, Tokyo, Japan).

### Animal experiments

These studies followed the Guide for the Care and Use of Laboratory Animals and were approved by the Ethics Committee of the People’s Hospital of Guangxi Zhuang Autonomous Region (approval no. KYGZR-2013-06, China). A total of 12 BALB/c-nu female mice (4–5 weeks old) were purchased from the Laboratory Animal Center of Guangxi Medical University (approval no. SCXK (Gui) 2014-0002, China) and housed in an specific pathogen free environment with sterilized water and feed. The nude mice were randomly divided into four groups (n = 3 in each group). NPC cells (1 × 10^6^) transfected with sh-miR-940, sh-Ctrl, LV-miR-940, or LV-Ctrl vectors were injected subcutaneously into the right axilla to establish subcutaneous xenografts. The mice were observed daily, and their general condition was recorded. The long and short tumor diameters were measured every 3 days. After 28 days, the mice were euthanized by cervical dislocation, and the tumor tissue was stripped and weighed. The tumor volume was calculated using the following formula: volume = length × width^2^ × 0.5 (mm^3^). One part of the grafted tumor specimens was stored at −80°C for Western blotting, and one part was fixed in formalin and later embedded in paraffin for H&E and CD34-PAS double staining.

### Hematoxylin–Eosin (H&E) staining

Paraffin-embedded nude mouse tumor tissue was cut to 4 μm thickness and heated at 65°C for 2 h. Sections were deparaffinized and rehydrated, and stained with a Hematoxylin and Eosin Staining Kit (Beyotime, Shanghai, China).

### CD34-Periodic acid Schiff (PAS) double staining

Qualitative and quantitative analysis of VM in nude mouse tumor xenograft tissue sections was detected by CD34-PAS assay [8]. The embedded nude mouse tissues were sectioned and then dewaxed, hydrated and antigenically repaired. Tissue specimens were blocked with goat serum and incubated overnight at 4°C with anti-CD34 primary antibody (1:200, #81289, Abcam, USA). Then, incubated with anti-mouse HRP-labeled polymer secondary antibody at 37°C for 30 min. Finally, DAB (Proteintech, China) was added for staining, followed by staining using the Glycogen PAS staining solution set (Dalian Meilun Biotechnology Co., Ltd., China). VM quantification was performed on at least three random regions using a fluorescence microscope DMi8 (Leica, Germany).

### Statistical analysis

All data are the mean ± standard deviation (SD) of three replicate experiments. Statistical analysis was performed using SPSS 21.0 (IBM, Chicago, USA) and GraphPad Prism 9 (San Diego, CA, USA). Student’s *t*-test was used to compare the means of the two groups, *p* < 0.05 was considered statistically significant.

## Results

### MiR-940 inhibits NPC cell proliferation, migration and tube formation in vitro

**Figure 1:**
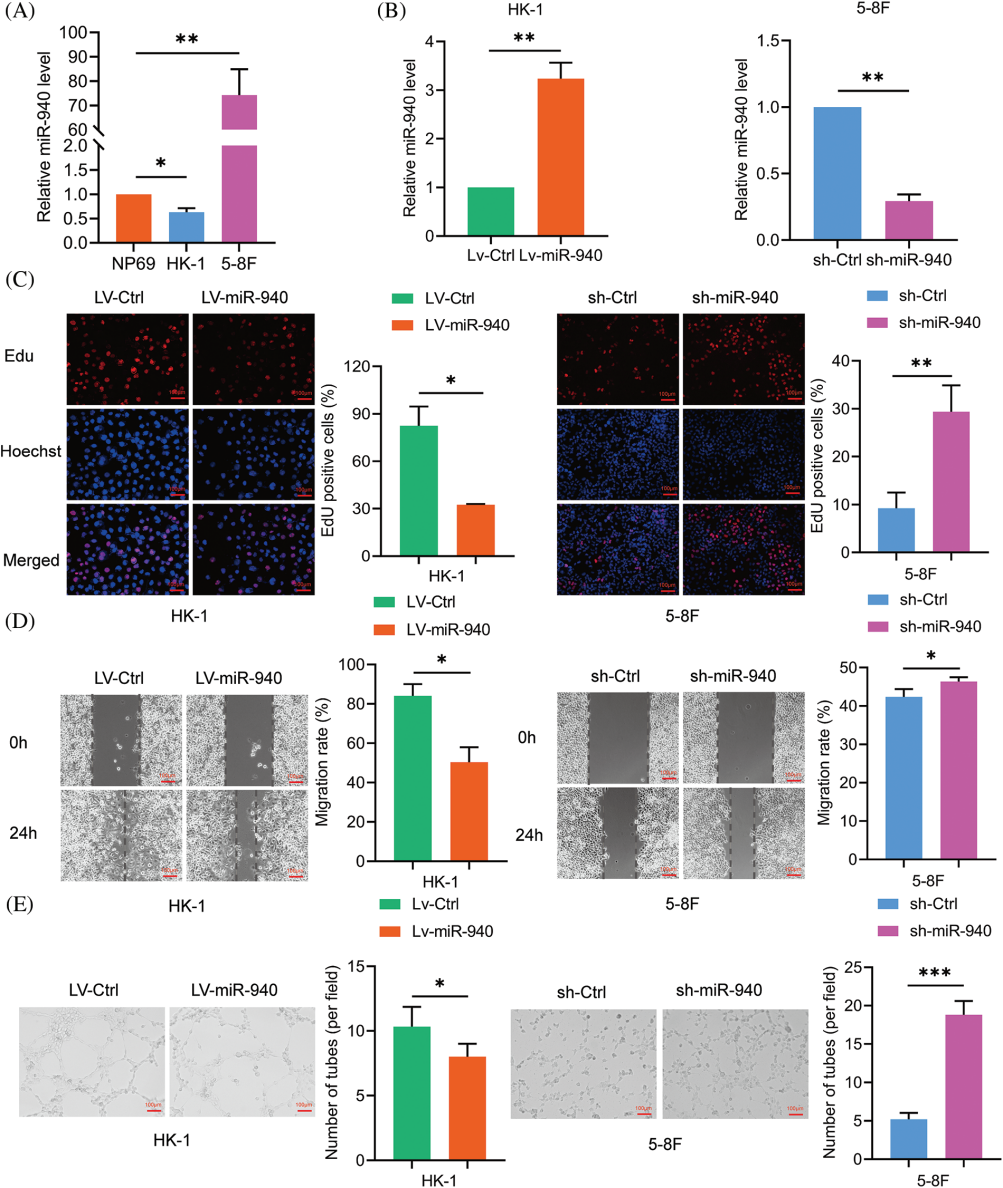
MiR-940 inhibits the malignant biological properties of NPC cells. (A) Relative expression of miR-940 in NP69 and NPC cells. (B) RT-qPCR analysis of LV-miR-940 lentivirus overexpression efficiency in HK cell lines and sh-miR-940 lentivirus knockdown efficiency in 5-8F cells. (C) EdU staining to assess NPC cell proliferation (×100). (D) Wound healing assay to assess the migration of NPC cells (×100). (E) 3D cell culture to detect VM channels (×100). Data are the mean ± SD from three experiments; *p < 0.05; **p < 0.01; ***p < 0.001.

Previous studies have alluded that miR-940 is downregulated in NPC patients compared to healthy individuals [18]. To investigate the biological effects of miR-940 in NPC, miR-940 expression was higher in the 5-8F cell line and lower in the HK-1 cell line compared to immortalized nasopharyngeal cells NP69 by RT-qPCR analysis (Fig. 1A). Then, miR-940 overexpressing lentivirus (LV-miR-940) and miR-940 knockdown (sh-miR-940) lentivirus were transfected into HK-1 cells and 5-8F cells, respectively. Fig. 1B shows the overexpression and silencing efficiency of miR-940. Edu assay revealed that overexpression of miR-940 significantly inhibited NPC cell proliferation, while knockdown of miR-940 promoted NPC cell proliferation (Fig. 1C). Wound healing assays indicated that the migratory ability of NPC cells was reduced when miR-940 was ectopically expressed and enhanced when miR-940 was downregulated (Fig. 1D). Meanwhile, 3D cells culture assay demonstrated that high miR-940 reduced tube formation while silencing of miR-940 showed the opposite effect (Fig. 1E). In conclusion, these results support that miR-940 inhibits the progression of NPC *in vitro*.

### ERBB2 is a target of miR-940 and correlates with poor prognosis in NPC patients

**Figure 2:**
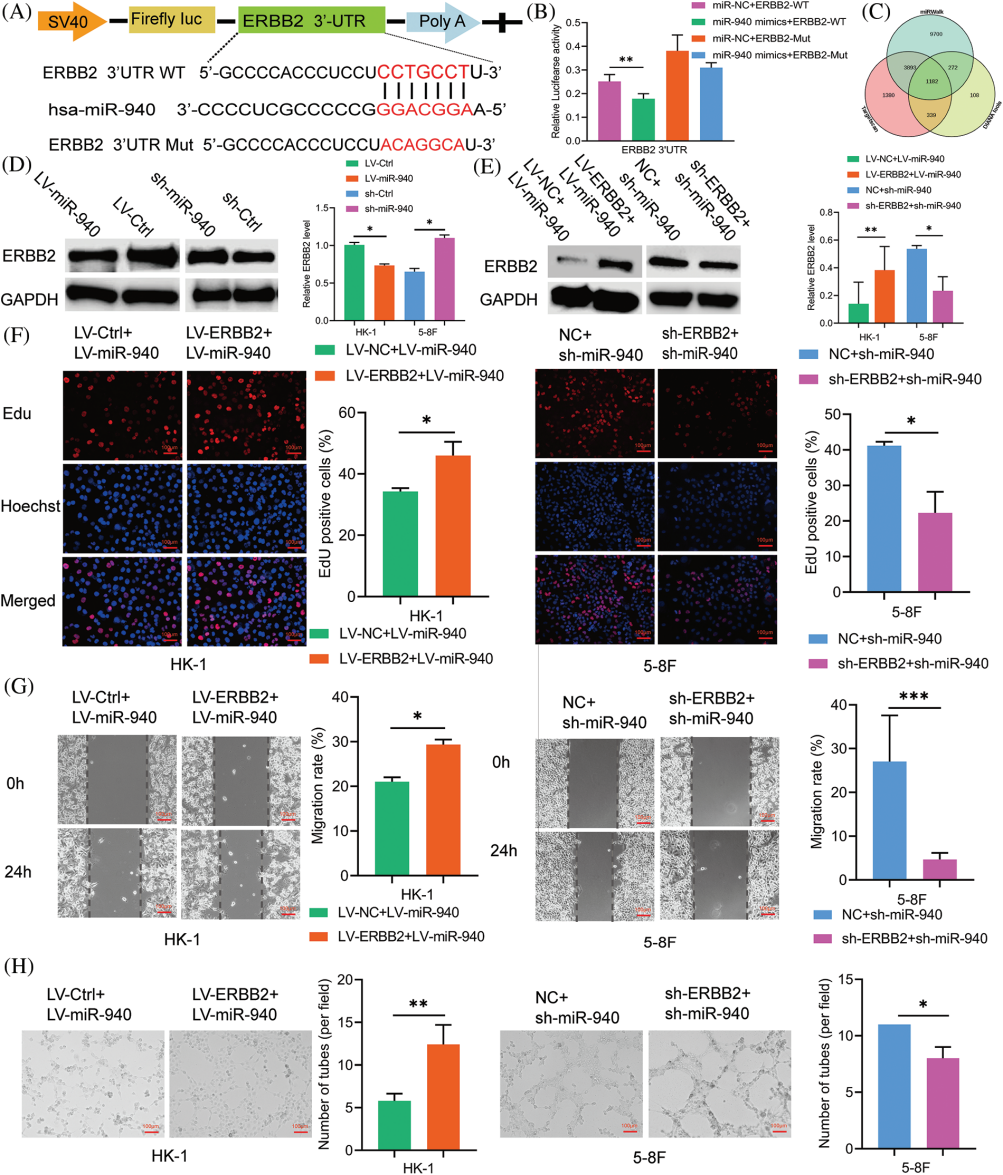
ERBB2 is a target of miR-940 and correlates with poor prognosis in NPC patients. (A) Diagram of the predicted miR-940 binding sequence on ERBB2 3′UTR. (B) Binding of miR-940 to ERBB2 3′UTR was verified by dual-luciferase reporter gene assay. (C) Overlapping of the target mRNAs of miR-940 based on TargetScan7.2, DIANA tools and miRWalk. (D and E) Expression of ERBB2 protein in miR-940 and rescue assays by western blotting. (F–H) 5-8F and HK-1 cells transfected with miR-940 and ERBB2 lentivirus were subjected to (F) EdU staining, (G) wound closure assay and (H) tube formation assay. Magnification × 100. Data are the mean ± SD from three experiments; *p < 0.05; **p < 0.01; ***p < 0.001.

To clarify how miR-940 functions, we used bioinformatics databases Targetscan7.2, DIANA tools and miRWalk to predict the target genes that miR-940 may directly regulate (Fig. 2A), and the results showed that ERBB2 had a stronger binding capacity to miR-940 (Fig. 2B). Next, we performed a dual luciferase reporter gene assay, and when cotransfected with miR-940 mimics, luciferase activity was significantly reduced in the ERBB2 wild type but not in the mutant group (Fig. 2C). In addition, western blotting showed that miR-940 upregulation decreased ERBB2 protein expression level in NPC, whereas miR-940 downregulation increased it (Fig. 2D). These results suggest that miR-940 regulates ERBB2 expression by silencing its mRNA.

**Figure 3:**
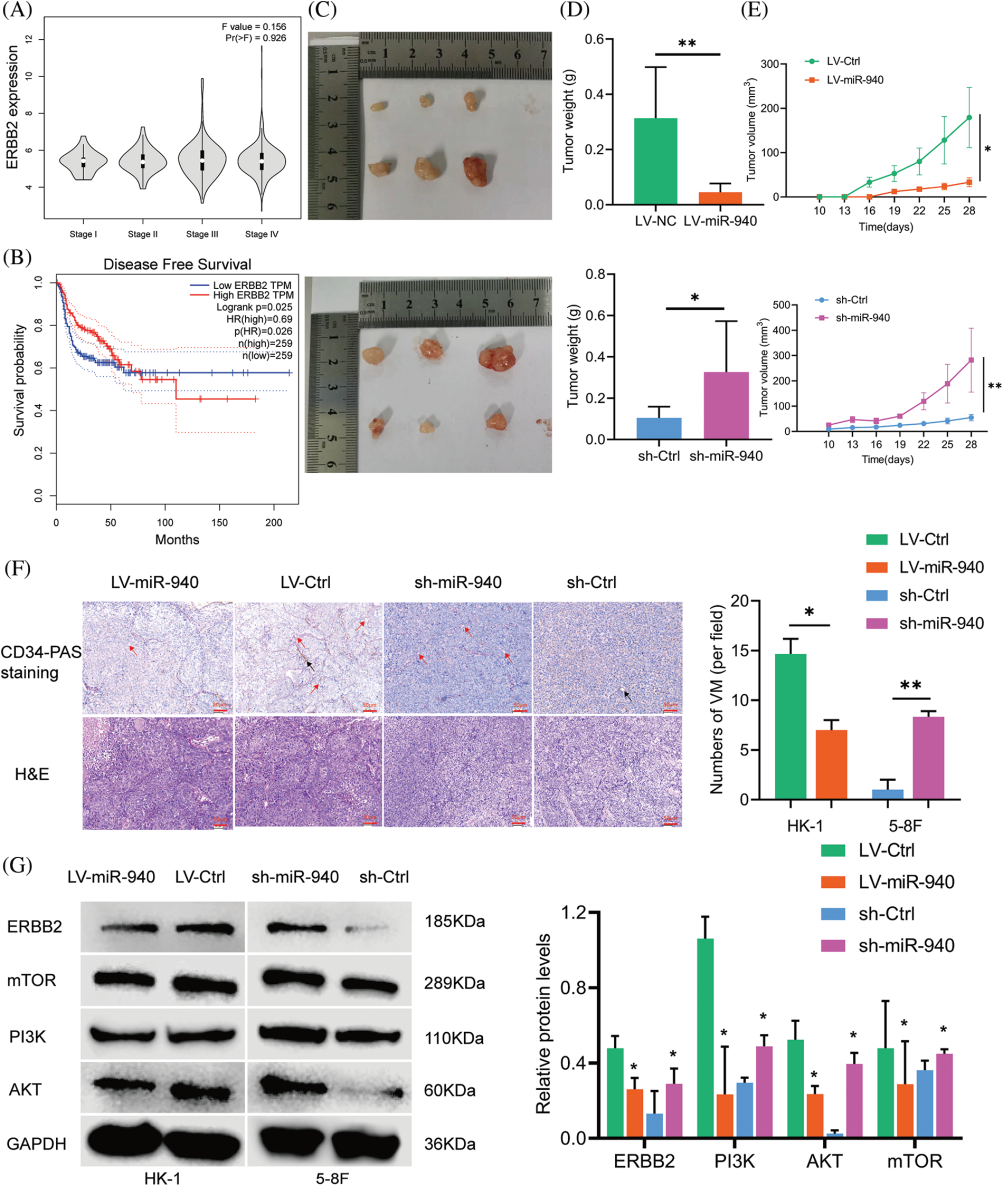
MiR-940 promotes NPC cell xenograft tumor growth and VM formation. (A) Expression levels of ERBB2 in NPC patients with stage I–IV from the GEPIA database. (B) The disease-free survival graph of NPC patients with high or low ERBB2 expression. (C) Images of excised xenograft tumors (after day 28). (D) Weight of tumors. (E) Growth curves of tumors. (F) H&E and CD34-PAS staining to detect tumor and VM channels in xenograft tumors. Red arrow = VM; black arrow = endothelial vasculature; magnification × 200. (G) Expression of ERBB2 and pathway-related genes in each group of xenograft tumors. Data are the mean ± SD from three experiments; *p < 0.05; **p < 0.01.

To further investigate whether miR-940 and ERBB2 are functionally relevant in NPC, we performed rescue experiments showing that upregulation of ERBB2 reversed the overexpression of miR-940 on proliferation, migration and VM formation in NPC cells, whereas downregulation of ERBB2 reduced the enhancement of proliferation, migration and VM formation induced by miR-940 silencing (Figs. 2F–2H). In addition, overexpression of ERBB2 attenuated the inhibition of ERBB2 expression by miR-940 overexpression, and silencing of ERBB2 counteracted the promotion of ERBB2 expression by miR-940 downregulation (Fig. 2E). Collectively, these data suggest that miR-940 exerts its function through ERBB2. To assess the clinical value of ERBB2 in NPC, we used the GEPIA database and noted that ERBB2 correlated with the clinical stage of NPC patients, with ERBB2 more likely to be highly expressed in patients with the higher stage (Fig. 3A). Also, Kaplan–Meier analysis showed that patients with high ERBB2 expression were more likely to have disease recurrence and poorer disease-free survival (DFS, *p* = 0.026; Fig. 3B).

### MiR-940 inhibits the growth and VM formation of NPC cell xenograft tumors

We investigated the role of miR-940 in NPC cells *in vivo* and inoculated HK-1 cells stably expressing LV-miR-940 and 5-8F cells with sh-miR-940 into female nude mice subcutaneously to construct a xenograft model, respectively. We observed that the miR-940 overexpression group had significantly smaller subcutaneous tumor weight and volume than the LV-Ctrl group, whereas the miR-940 knockdown group had a larger tumor weight and volume than the sh-Ctrl group (Figs. 3C–3E). H&E staining showed that miR-940 overexpression reduced tumor cells (Fig. 3F). CD34-PAS double staining confirmed that miR-940 ectopic expression mice had significantly fewer VM channels compared to controls, whereas miR-940 knockdown formed more VM channels (Fig. 3F). Western blotting showed that ERBB2 expression was reduced in the miR-940 overexpression group, whereas the opposite result was observed in the miR-940 knockdown group. In conclusion, the above results indicated that miR-940 expression inhibited NPC proliferation, migratory and VM formation.

### MiR-940 inhibits NPC progression via PI3K/AKT/mTOR signaling axis

ERBB2/PI3K/AKT/mTOR signaling has been reported to mediate biological behaviors such as cell proliferation, differentiation and angiogenesis [28,31,35]. Next, we explored the effect of miR-940 on ERBB2/PI3K/AKT/mTOR signaling in NPC. As shown in Fig. 3G, ERBB2 was downregulated in response to miR-940 overexpression, and PI3K, AKT and mTOR expression levels were also decreased. MiR-940 downregulation increased the expression level of ERBB2, whereas PI3K, AKT and mTOR levels were also increased. In addition, western blotting analysis of xenograft tissue proteins also confirmed this effect (Fig. 3G). This suggested that miR-940 negatively regulated molecules on the PI3K/AKT/mTOR signaling axis by suppressing ERBB2 expression, thereby inhibiting the progression of NPC.

### CircMAN1A2 is a potential regulator of miR-940

**Figure 4:**
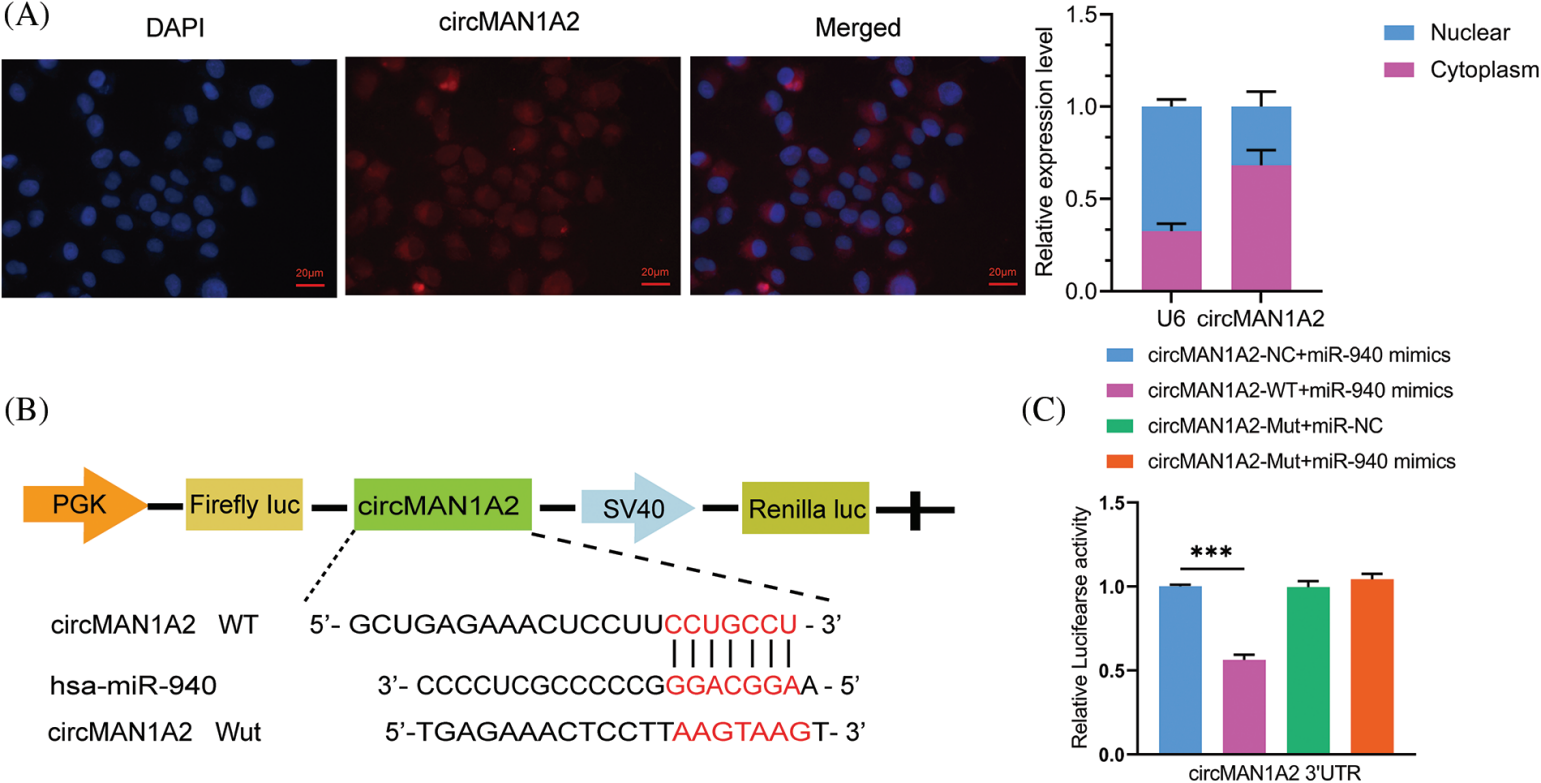
CircMAN1A2 is a potential regulator of miR-940. (A) FISH reveals the subcellular localization and expression of circMAN1A2 in 5-8F cells (×400). (B) Potential binding sites for MiR-940 and circMAN1A2 3′UTR-WT. (C) CircMAN1A2 binding to miR-940 was verified by dual luciferase reporter gene assay. Data are the mean ± SD from three experiments; ***p < 0.001.

According to the competing endogenous RNAs (ceRNAs) hypothesis [36], circRNAs may be involved in disease progression by acting as miRNAs sponges to promote the expression of target genes, which inspired us to explore whether miR-940 is regulated by specific circRNAs. First, we used the circNET database to predict circRNAs that might bind to miR-940, and the results showed that circMAN1A2 had binding sequences to miR-940. To confirm the prediction, RNA-FISH analysis demonstrated that circMAN1A2 was primarily localized in the cytoplasm, and it was tentatively determined that circMAN1A2 played the role of ceRNA (Fig. 4A). In addition, studies revealed that circMAN1A2 is highly expressed in the serum and tissues of NPC patients [23]. Dual luciferase assay analysis revealed that miR-940 mimics significantly reduced the luciferase activity of the circMAN1A2 3′UTR-WT reporter gene (Figs. 4B and 4C), whereas no change was observed in circMAN1A2 3′UTR-Mut and 3′UTR-NC. Taken together, our data showed that circMAN1A2 had miRNA binding sites and acted as a sponge for miR-940.

### CircMAN1A2 accelerates malignant phenotype in NPC cells

**Figure 5:**
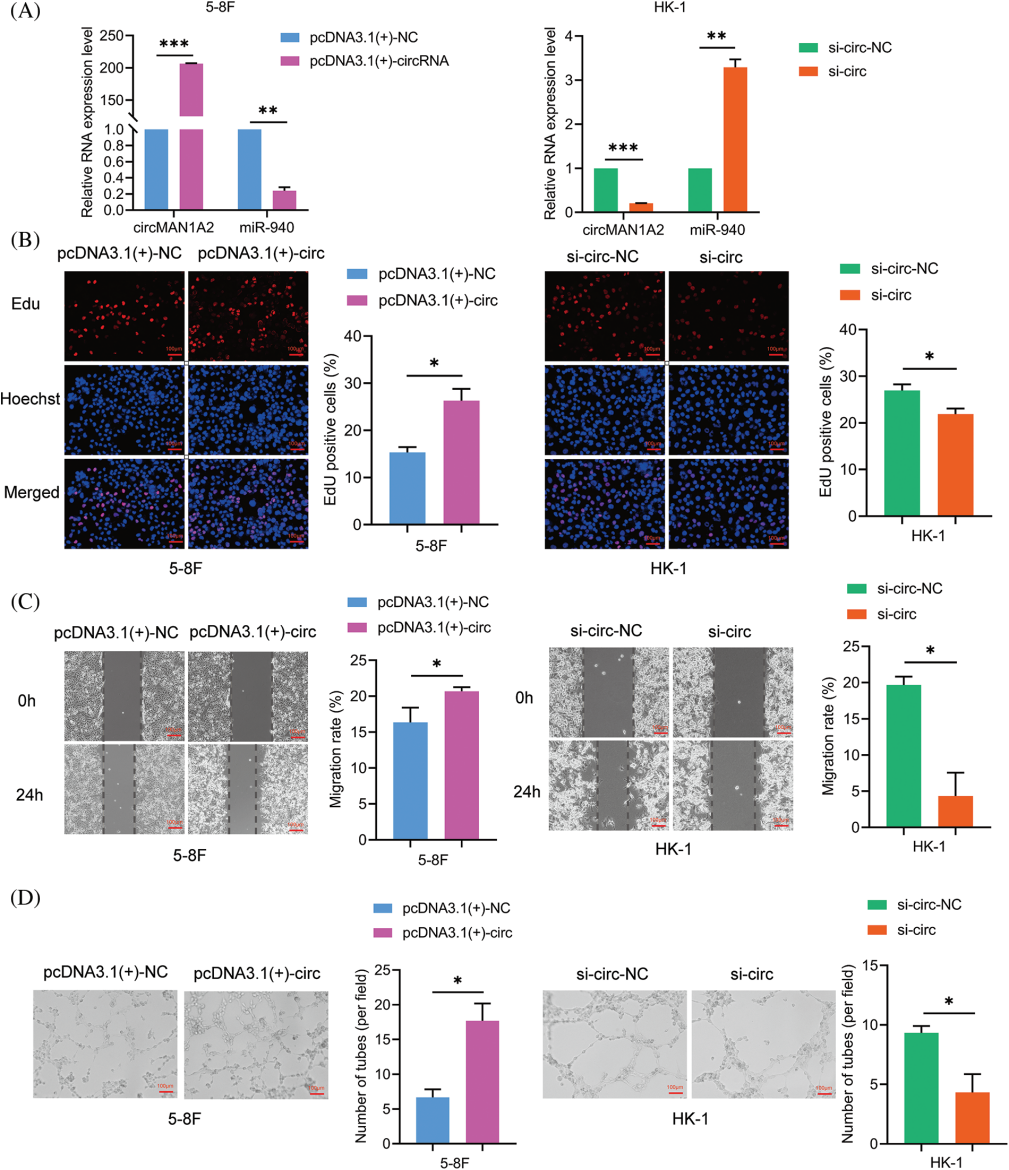
CircMAN1A2 promotes the malignant behavior of NPC cells. (A) Relative expression of circMAN1A2 and miR-940 in NPC cells after circMAN1A2 overexpression or silencing. (B–D) Determination and quantification of (B) EdU staining, (C) wound healing assay and (D) tube formation in circMAN1A2 overexpressed or knockdown NPC cells. Magnification × 100. Data are the mean ± SD from three experiments; *p < 0.05; **p < 0.01; ***p < 0.001.

To verify the function of circMAN1A2 in NPC cells. We characterized the oncogenic phenotype in 5-8F cells with circMAN1A2 overexpression (pcDNA3.1(+)-circMAN1A2) and in HK-1 cells with circMAN1A2 silencing (si-circMAN1A2). RT-qPCR showed that pcDNA3.1(+)-circMAN1A2 significantly upregulated the expression of circMAN1A2, and si-circMAN1A2 significantly down-regulated it (Fig. 5A). Functionally, overexpression of circMAN1A2 promoted NPC cell proliferation, migration and VM channels *in vitro*, and silencing circMAN1A2 had the opposite effect (Figs. 5B–5D). In addition, the upregulation of circMAN1A2 significantly reduced miR-940 in NPC cells, whereas the deletion of circMAN1A2 promoted it (Fig. 5A). Thus, circMAN1A2 promoted the malignant behavior of NPC cells.

### CircMAN1A2 promotes the VM of NPC cells by attenuating the inhibitory effect of miR-940 on ERBB2

**Figure 6:**
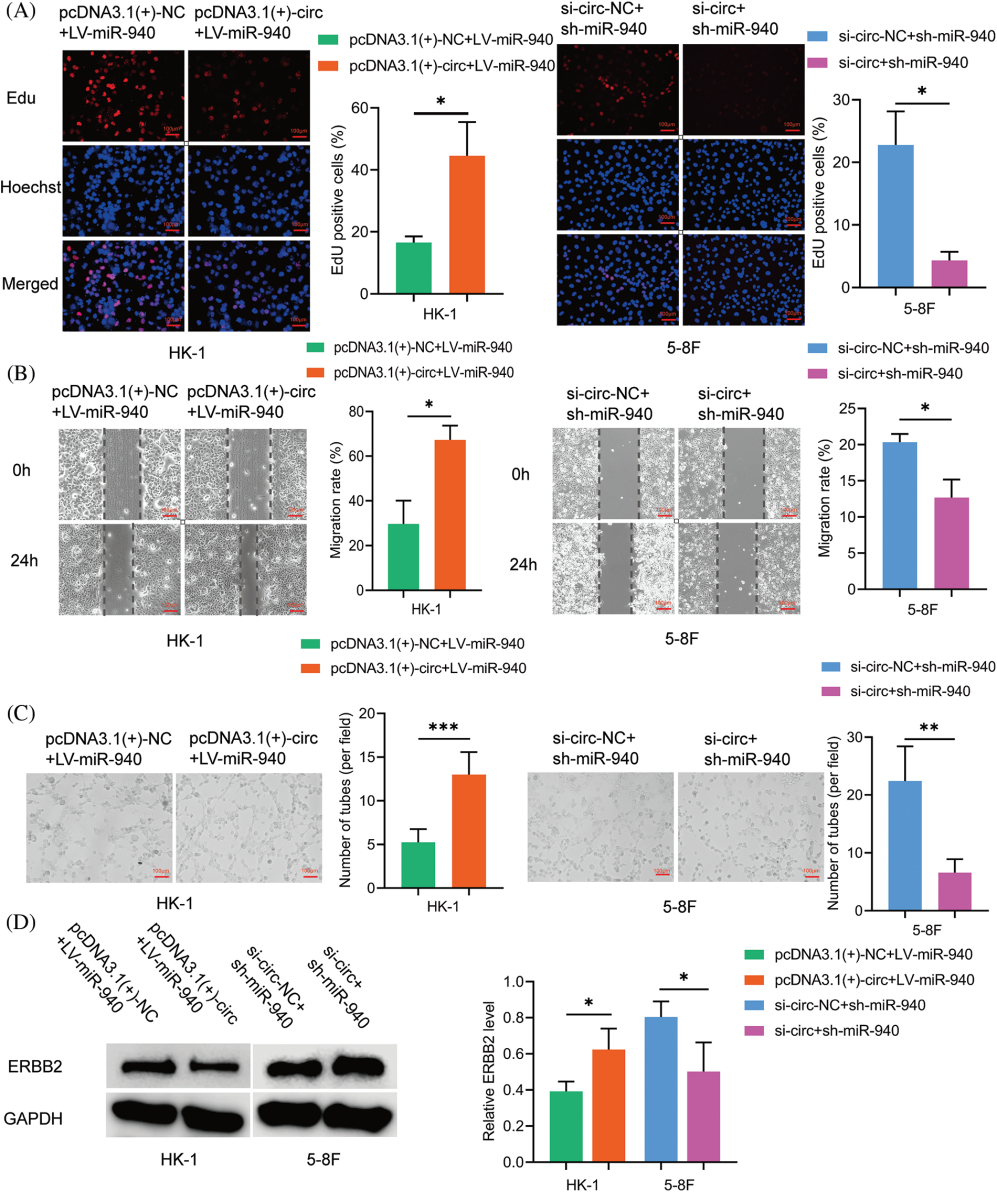
CircMAN1A2 promotes NPC progression by sponging miR-940. (A–C) The effect of CircMAN1A2 and/or miR-940 expression on NPC cells by (A) EdU staining, (B) wound healing assay, and (C) tube formation assay. Magnification × 100. (D) The effect of circMAN1A2 and/or miR-940 on ERBB2 expression was examined by western blotting. Data are the mean ± SD from three experiments; *p < 0.05; **p < 0.01; ***p < 0.001.

To clarify whether circMAN1A2 plays a role in promoting NPC cells by sponging miR-940, pcDNA3.1(+)-circMAN1A2 or si-circMAN1A2 were transfected on the basis of miR-940 overexpression and silencing of NPC cells. Rescue experiments showed that high expression of circMAN1A2 counteracted the inhibitory ability of miR-940 overexpression on NPC cell proliferation, migration and tube formation. Conversely, si-circMAN1A2 eliminated the promoting effect of miR-940 silencing (Figs. 6A–6C). In addition, reduced ERBB2 protein expression after silencing circMAN1A2 could be reversed by the downregulation of miR-940. Conversely, high expression of circMAN1A2 promoted ERBB2 expression, which could be rescued by miR-940 expression (Fig. 6D). In summary, our findings suggested that circMAN1A2 may be an oncogenic factor in NPC cells that promotes ERBB2 expression via miR-940.

## Discussion

**Figure 7:**
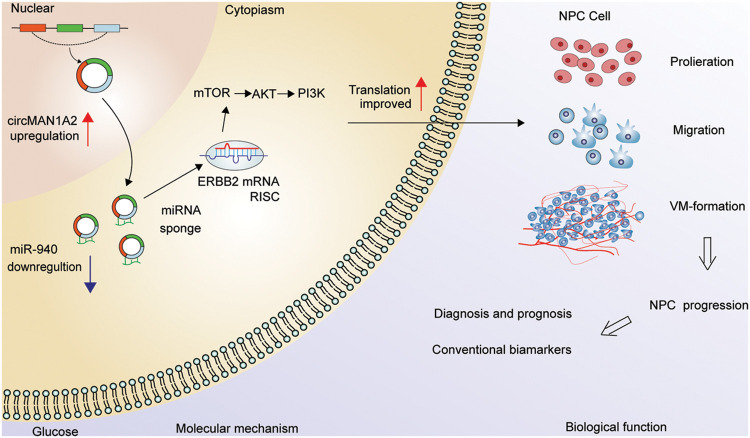
Hypothetical model of circMAN1A2 function in NPC. In the nucleus, circMAN1A2 is formed by reverse splicing. Upon export to the cytoplasm, circMAN1A2 acts as a sponge for miR-940, promoting ERBB2 expression and further activating PI3K/AKT/mTOR signaling in NPC progression.

NPC patients with VM were more likely to develop metastases or have poorer survival outcomes [37–39]. As there is no effective clinical strategy for VM, there is an urgent need for new targets with VM-blocking effects to improve the treatment outcome of NPC patients. In our study, we first demonstrate that miR-940 is a key miRNA involved in VM formation and progression in NPC. Second, miR-940 overexpression inhibits VM generation in NPC cells by competitively binding to ERBB2, while high ERBB2 may predict clinical prognosis. Third, circMAN1A2 is identified as an adsorption sponge for miR-940 to promote NPC genesis and progression, and its enormous potential as a molecular marker and target for tumor angiogenesis is explored (Fig. 7).

In this study, we note a miR-940 that is significantly downregulated in NPC, which was dysregulated in solid tumors and is a tumor suppressor [11]. Functionally, miR-940 overexpression inhibited NPC cell proliferation, migration and VM channel *in vitro* and *in vivo*, whereas miR-940 knockdown has the opposite effect. It was well known that miRNAs control the post-transcriptional expression levels of genes [40]. We prove that ERBB2 is a direct target of miR-940 based on bioinformatic analysis and luciferase reporter gene experiments, and ERBB2 was considered to be an oncogene active in almost all cancers studied [27]. Here, silencing ERBB2 rescues the ability of miR-940 low expression to promote cell malignancy, and overexpression of ERBB2 also altered the effect of miR-940 on NPC function. Furthermore, we found through the GEPIA database that increased ERBB2 expression correlates with tumor stage and predicted a poor prognosis in NPC patients. All these results suggest that miR-940 regulates the progression of NPC by suppressing ERBB2 expression.

There is increasing evidence that circRNAs can perform various biological functions as ceRNAs in combination with miRNAs, where circRNAs in different subcellular compartments can perform different functions [41]. In our study, FISH and luciferase reporter gene analysis consistently show that circMAN1A2 binds directly to miR-940, and circMAN1A2 has been reported to be highly expressed in NPC [23]. Furthermore, upregulation of circMAN1A2 promotes NPC cell proliferation, migration and VM, while silencing circMAN1A2 has the opposite effect. In addition, circMAN1A2 reversed the effect of miR-940. All the above data imply that circMAN1A2 promotes VM formation in NPC cells by sponging miR-940.

Aberrant activation of PI3K/Akt/mTOR signaling pathway is considered one of the major factors in the development of many cancers [42]. Several studies have shown that excessive accumulation of ERBB2 is one of the key triggers of abnormal excitation of the PI3K/Akt/mTOR signaling axis [43] and is involved in the regulation of VM formation in cancer cells [32]. Our data reveals that miR-940 overexpression reduces ERBB2 and PI3K/Akt/mTOR expression, whereas the knockdown of miR-940 resulted in their increase. In addition, ERBB2 reversed the regulation of PI3K/Akt/mTOR expression by miR-940. More importantly, circMAN1A2 reversed the effect of miR-940 on ERBB2 expression and cell phenotype. Thus, miR-940/ERBB2/PI3K/Akt/mTOR is a downstream target of circMAN1A2 in regulating NPC cogenesis and VM formation.

In cancer therapy, drugs can be used to block several cancer-related signaling pathways or to introduce circRNAs *ex vivo* [44]. Currently, antisense oligonucleotides (ASOs) and siRNAs are commonly used as a tool for regulating gene expression due to their high specificity and safety [45]. Several RNA-based drugs (e.g., ASO) have received FDA approval and many are in clinical evaluation [46–48], paving the way for circRNA-based therapies. In fact, circRNA’s unique loop structure makes it more stable than mRNA or miRNA and thus superior to conventional linear oligo antisense oligonucleotides nucleotides as a molecular therapeutic target against tumors [49], and targeting circRNA may be a promising approach to inhibit tumor progression [50]. In the future, the synergy between conventional anti-angiogenic therapy and targeting VM-related circRNA may complement new anti-angiogenic therapeutic strategies for cancer, which broadens our understanding of cancer treatment. Of note, we found that circMAN1A2 acts directly on VM generation. Therefore, circMAN1A2 may be a promising target for anti-VM therapy in NPC patients.

However, our study also has limitations. In future studies, assessing the clinical value of circMAN1A2 needs to include sufficient follow-up data from NPC samples, which is important for subsequent clinical translation. Second, researchers confirmed that an effective anti-angiogenic treatment strategy should inhibit both VM and EDV [51]. Next, the feasibility of combined VEGF and ERBB2 therapy will be measured to provide a new treatment strategy for advanced or metastatic NPC.

In conclusion, the present study suggests that circMAN1A2 is associated with the VM of NPC. CircMAN1A2 promotes NPC progression through its function as a ceRNA, spongy miR-940 regulation of downstream ERBB2 expression, and activation of PI3K/AKT/mTOR pathway (Fig. 7). These will provide potential targets for understanding the progression, diagnosis and treatment of NPC.

## Data Availability

All data generated or analyzed during this study are included in this published article.
